# Photothermal Effect of 970 nm Diode Laser Irradiation on *Enterococcus faecalis* Biofilms in Single-Rooted Teeth Ex Vivo

**DOI:** 10.3390/dj12100308

**Published:** 2024-09-27

**Authors:** Soraya Tanner, Anna Thibault, Julian Grégoire Leprince, Serge Bouillaguet

**Affiliations:** Division of Cariology and Endodontology, CUMD–University Clinics of Dental Medicine, University of Geneva, 1205 Geneva, Switzerland; anna.thibault@unige.ch (A.T.); julian.leprince@unige.ch (J.G.L.); serge.bouillaguet@unige.ch (S.B.)

**Keywords:** *Enterococcus faecalis*, endodontic disinfection, 970 nm diode laser, root canal therapy

## Abstract

**Objective**: The aim of this study was to evaluate the photothermal effect of a 970 nm diode laser on *Enterococcus faecalis* biofilms. **Methods**: 72 extracted human single-rooted teeth were prepared, sterilized, and inoculated with *Enterococcus faecalis* to establish a two-week-old biofilm. The specimens were divided into six groups (n = 12): Group 1 (G1)—negative control (PBS—no laser), Group 2 (G2)—positive control (1% NaOCl rinse—no laser), Group 3 (G3)—a 970 nm laser in 1.5 W pulse mode, Group 4 (G4)—a 970 nm laser in 2 W pulse mode, Group 5 (G5)—a 970 nm laser in 1.5 W continuous mode, Group 6 (G6)—a 970 nm laser in 2 W continuous mode. Bacterial viability was evaluated using the LIVE/DEAD BacLight kit and analyzed by flow cytometry (FCM). Temperature changes on the root surface during irradiation were analyzed using a K-type thermocouple. Data were statistically analyzed using one-way ANOVA and Tukey’s multiple comparison test (α = 0.05). **Results**: Bacterial viability was significantly reduced after laser irradiation in continuous mode using 1.5 W (21% of live bacteria) and 2 W (14% of live bacteria). When the pulsed mode was applied, the reduction in bacterial viability was less, with a mean survival of 53% (1.5 PF, whereas 29% of bacteria survived after 2 W irradiation). **Conclusions**: The 970 nm diode laser at 2 W continuous mode effectively reduced the viability of *E. faecalis* biofilms in root canals without causing unacceptable temperature rises at the root surface.

## 1. Introduction

The treatment of necrotic teeth requires the elimination of microorganisms that form a bacterial biofilm adherent to the root canal walls, then the root canal space is filled to avoid subsequent reinfection [1,2]. The treatment involves three critical steps known as shaping, disinfection, and filling. Mechanical shaping using endodontic files enables the elimination of infected tissues from the main canal(s), the debridement of biofilm from the root canal walls and the enlargement of the root canal to facilitate the penetration of irrigating solutions to the apical portion of the root canal space [3]. The chemical effect of the mainly used solutions, i.e., sodium hypochlorite (NaOCl) and ethylenediamine tetra acetic (EDTA), complements the mechanical action of shaping instruments during disinfection [4].

NaOCl is a strong antimicrobial agent which—among other actions—also has the advantage over other irrigants of dissolving organic tissues, whereas EDTA acts as a chelating agent for calcium ions, dissolving inorganic debris and eliminating the smear layer formed during the shaping of the root canal [5,6]. However, even though these two irrigant agents are the two most recommended, it is known that hypochlorite accidents can occur during endodontic treatment, resulting in the extrusion of NaOCl beyond the apex. This can lead to significant tissue damage, as contact between hypochlorite and vital tissues may cause ulceration, hemolysis, and, in some cases, scarring or nerve injury [7]. This may lead to looking for alternative strategies to eradicate bacteria.

While it is not straightforward to determine the bacterial load threshold under which a favorable treatment outcome can be expected [8,9,10], it has been clearly demonstrated that it should be reduced as much as possible to increase the probability of treatment success [8,9]. Both the instrumentation and disinfection of the root canal contribute to a decrease in bacterial load within the canal [11], but it is extremely challenging, if at all possible, to remove all bacteria and their derivates from a contaminated root canal system [12]. A substantial percentage of the root canal walls remains unaffected by rotary instruments, thereby leaving a substantial amount of potentially infected dentin [13]. These untouched dentin areas may harbor bacteria that diffuse into dentin tubules, deep within the dentin, up to 1150 μm [14]. The complex structure of root canal systems, which often include fins, deltas, isthmus, and lateral canals, adds complications to the process of achieving a complete disinfection. Bacteria in these areas remain protected from the direct action of disinfectants, and irrigating solutions have limited penetration into dentin [15,16]. Also, there is evidence showing that several pathogens, such as *E. faecalis*, are harder to eliminate from infected root canals [17]. This bacterium can survive extremely harsh environments, even under starvation conditions. Its ability to form strong biofilm on root canal walls and to deeply colonize dentin tubules, without requiring synergistic support from other bacteria, makes it highly resistant to conventional antimicrobial treatments such as CaOH_2_ and a particularly challenging pathogen to eradicate [18,19,20].

For this reasons, various alternative strategies including laser irradiation have been proposed to optimize endodontic disinfection [21].

Lasers can be used to directly irradiate root canals or to activate photoactive substances (PAD—photoactivated disinfection) and irrigating solutions (LAI—Laser-Activated Irrigation, PIPS-SWEEPS—Photon-Induced Photoacoustic Streaming; Shock Wave-enhanced emission photoacoustic streaming) [22].

PAD targets photoactive molecules (photosensitizers) that produce reactive oxygen species with antibacterial action upon light irradiation (photochemical effect). The combination of red lasers (630–700 nm), and methylene blue has shown promise against cariogenic, endodontic, and periodontal bacteria [23,24,25,26]. LAI and PIPS-SWEEPS target the water content of irrigating solutions where cavitation bubbles are produced upon irradiation, and PIPS/SWEEPS combine the laser-induced cavitation with shock-wave enhancement [27,28,29]. Laser-induced photoacoustic waves produced by Er:YAG lasers (2940 nm) were shown to promote fluid streaming inside root canals [27,28,30] and to remove bacterial biofilms [31]. However, there are concerns about the risks of irrigant extrusion when using LAI and PIPS-SWEEPS, mostly because NaOCl extruded past the root apex may induce tissue necrosis [32,33,34]. Also, the cost of these instruments remains elevated.

More recently, near-infrared diode lasers (810–980 nm), have been suggested as an alternative for endodontic disinfection among other laser types. These wavelengths, which are strongly absorbed by water and dental hard tissues, have the potential to reach deeper layers of dentin where the heat generated (photothermal effect) can contribute to the elimination of microorganisms. These lasers can be coupled to thin (200 μm in diameter) conducting fibers, which are directly applied inside the root canal. Previous reports have shown promising antibacterial activity against several endodontic bacteria [35,36,37], whereas other studies report no noticeable effect on the bacterial viability [38,39,40,41,42]. Further, the risk of thermal injury to periodontal tissues after long and continuous exposure to NIR laser irradiation has been advocated [43,44]. Borges et al. found that a 970 nm diode laser in continuous mode only reduced the viability of *E. faecalis* biofilms grown on dentin blocks by 68% [45]. Schoop et al. found that, above a power of 1.5 W, the increase in bactericidal effect was also accompanied by changes in the surface of slices of root dentin after irradiation [37]. Alfredo et al. 2008 reported a 12 °C temperature rise when root canals were irradiated with 5.0 W [46], underlining the need to carefully control the laser parameters to balance the antimicrobial activity with the risks of adverse effects to surrounding tissues [47,48]. It is therefore hypothesized that pulsed waves (PW) of a near-infrared 970 nm laser wavelength, which can be used to modulate the total energy applied, may be more appropriate for the elimination of bacteria with limited risks of adverse thermal effects.

The purpose of this study was, therefore, to assess the antibacterial effect of a 970 nm diode laser against *Enterococcus faecalis* biofilms using continuous-wave (CW) and pulsed-wave (PW) modes, with a power output ranging from 1 to 2 W, while monitoring temperature changes inside root canals during laser irradiation. The hypothesis tested in the current study was that the irradiation of NIR lasers, which can penetrate 1 mm deep inside dentin tissues, might reach the areas untouched by irrigants and instruments, thereby providing a complementary advantage in terms of root canal disinfection [49].

## 2. Materials and Methods

In this current study, a biofilm model that was previously described by Provoost et al. 2022 was employed [50].

### 2.1. Teeth Selection

Seventy-two single-rooted human teeth were collected after extraction at the University clinics of dental medicine, University of Geneva, Switzerland. In accordance with the Swiss Federal Act on Research involving Human Beings, ethical approval is not necessary, since the use of anonymized extracted teeth for research purposes falls outside the scope of the legislation (https://www.fedlex.admin.ch/eli/cc/2013/617/en accessed on as 1 September 2023).

### 2.2. Specimen Preparation

Teeth were cleaned from external tissue remains and access cavities were opened. To measure the root canal length, a #10 K-File was inserted into the canal until the end of the file emerged through the major apical foramen, as observed under a stereomicroscope (Leica MZ6 Leica, Heidelberg, Germany). The working length was set 1 mm shorter and teeth were mechanically enlarged to an apical diameter ISO 030 using ProTaper Gold rotatory files (Dentsply Sirona Endodontics, Ballaigues, Switzerland). A 3% NaOCl solution was used during shaping and the final irrigation was performed using 17% EDTA, which was finally neutralized with normal saline. Each crown was cut using a water-cooled diamond fissure bur (NeoDiamond, Kennesaw, GA, USA) to obtain a 12-mm-long root segment. Root segments were sonicated 2 × 15 min (Vevor, Digital Ultrasonic Cleaner, Nanjing, China) in distilled sterile water, then placed in a thick-walled transparent silicone tube (Semadeni, Ostermundigen, Switzerland) into which distilled water was added, and then sterilized by autoclaving at 121 °C for 40 min (Tomy, SX-500E, Tokyo, Japan).

### 2.3. Bacteria

A 50 μL suspension of *E. faecalis* (HUG 135737) from frozen stocks was cultured in 5 mL of BHI broth (BHI, Becton Dickinson, Allschwil, Switzerland) and incubated at 37 °C for 4 h. The bacterial concentration was adjusted to an OD_600nm_ = 0.2 (~3.0 × 10^7^ bacteria/mL, Biowave II, Biochrom WPA, Cambridge, UK, CG) [50] and the solution injected into the silicone tubing containing 6 roots.

**Figure 1:**
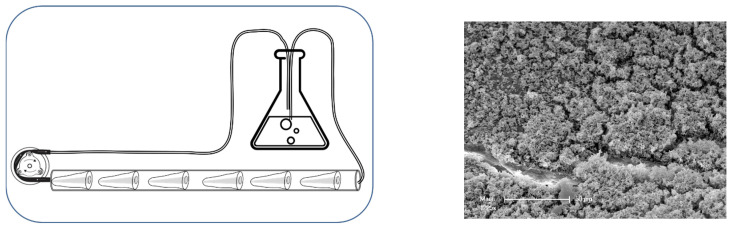
Bacterial biofilms were grown for 2 weeks. In order to eliminate dead bacteria and debris, the bacterial culture was passed through a peristaltic pump and refreshed at regular intervals. Scanning electron microphotography of the bacterial biofilm grown on dentin root canals (mag. ×1200).

The tube was connected to an airtight Erlenmeyer flask on one side and to a peristaltic pump on the other, thus producing a closed circuit (Figure 1). The silicone tube was installed in an incubator (HeraCell 150, Thermo Fisher Scientific, Inc., Reinach, Switzerland) at 37 °C.

With a flow rate of at least 100 μL/min, the bacterial culture was circulated twice a day for 48 h, after which it was replaced with fresh BHI and recirculated twice a day for one week. Over the next week period, phosphate-buffered saline (PBS, Gibco, Thermo Fisher Scientific Inc., Reinach, Switzerland) was introduced in the circuit and circulated twice a day, for 2 min. Finally, fresh PBS was used to eliminate dead bacteria and debris and the peristaltic pump disconnected. The 6 roots were separated by cutting the silicone tube 5 mm above the coronal access of every root.

The apex of each root was wiped with sterile gauze Impregnated with sodium hypochlorite and sealed with a light-body silicon material, to avoid bacterial loss during and after laser treatment.

### 2.4. Groups

Teeth were randomly selected from a jar containing all specimens. This approach was considered appropriate, since standardized, single-rooted teeth were used. The specimens were divided into six groups, two control and four experimental ones, the latter exposed to laser irradiation with various parameters (see below). The control groups consisted of either a mere PBS rinse, or an irrigation with 3 mL of 1% NaOCl, without laser use. For experimental groups, a 970 nm wavelength of the SIROLaser Blue coupled to the 200 μm EasyTip Endo (both from Sirona Dental System GmbH, Bensheim, Germany) was used to irradiate root canals. Each root canal was treated for 80 s. The EasyTip Endo fiber was inserted to the working length and then retracted in the coronal direction with a helical movement, to increase the area exposed. The laser settings used were as follows: 1.5 W in pulsed-wave mode, 1.5 W in continuous-wave mode, and 2 W in the pulsed- and continuous-wave modes. All experiments were repeated twice by the same operator, who had undergone laser training from the manufacturer. After irradiation, root canal content was collected using a manual #30 K-File (Dentsply Maillefer, Ballaigues, Switzerland) that was employed to scrub the dentin wall for 30 s. A total of 1 mL of phosphate-buffered saline (PBS) solution at pH 7.4 was used to irrigate the root canals, and the solution was then collected into a sterile tube along with the K-File. The tube was sonicated for 20 s (Vortex-Genie 2, Scientific Industry, Bohemia, NY, USA) to detach bacterial aggregates from the K-File and to make single-cell suspensions [51].

Bacterial viability was assessed using the LIVE/DEAD BacLight Kit (Life Technologies Europe BV, Zug, Switzerland), which employs two fluorescent dyes—SYTO-9 (green) and propidium iodide (red)—to evaluate cell membrane integrity. Flow cytometry was conducted using a BD Accuri C6 flow cytometer (BD Accuri Cytometers, Ann Arbor, MI, USA) equipped with a 488 nm laser for the excitation of both dyes. SYTO-9 fluorescence was detected in the FL1 channel (BP 533/30), while propidium iodide fluorescence was captured in the FL3 channel (LP > 670) [52]. Data analysis was performed using FlowJo software (FlowJo for Windows, version 10.0.06, 2014, Tree Star Inc., Ashland, OR, USA), and results were reported as the percentage of viable cells relative to the total number of cells in the suspension.

### 2.5. Temperature

**Figure 2:**
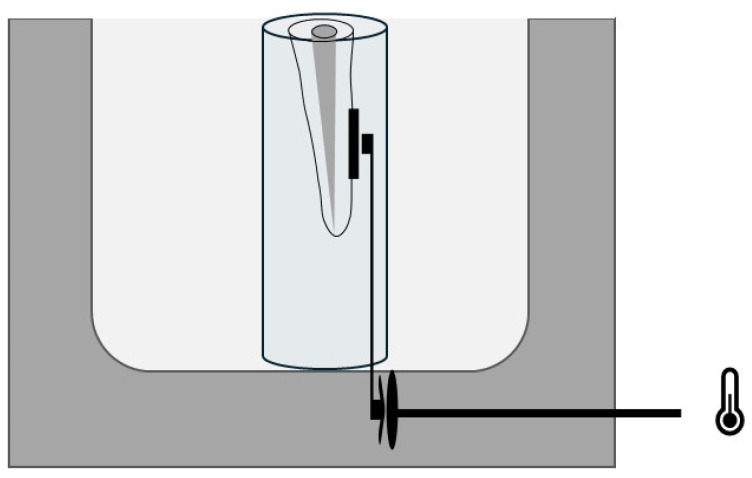
Illustration of the experimental configuration for heat control in the root canal, showing thermocouple placement in the apical third. The thermocouple was connected to a thermometer that displayed the temperature directly on the screen. The temperature was recorded every 5 s, until returning to 37 °C.

For temperature measurements, root canals were immersed in a narrow polyethylene tube filled with water, which was introduced in a water bath with a constant temperature of 37 °C (Grant Instrument, Optima T100, Cambridge, UK). This experimental setup was designed to keep the root hydrated during laser irradiation and to simulate in vivo thermal conductivity as effectively as possible. The thermal conductivity values of the tube/water system (0.6 W/m K) approached that of bone (0.58–1.2 W/m K). A thermocouple (B+B, Thermo-Technik GmbH, Germany) was used to measure the temperature on the root surface to the apical portion of the root; it was recorded every 5 s, until returning to 37 °C (Thermometer 306, Voltcraft, Germany). The setup is illustrated in Figure 2.

Data were statistically evaluated using one-way ANOVA and Tukey’s multiple comparison test (*p* = 0.05) in IBM SPSS Statistics 27.0.1.0 (Academic authorized user, 5725-A54). Each experiment was performed in triplicate and repeated four times (n = 12).

## 3. Results

**Figure 3:**
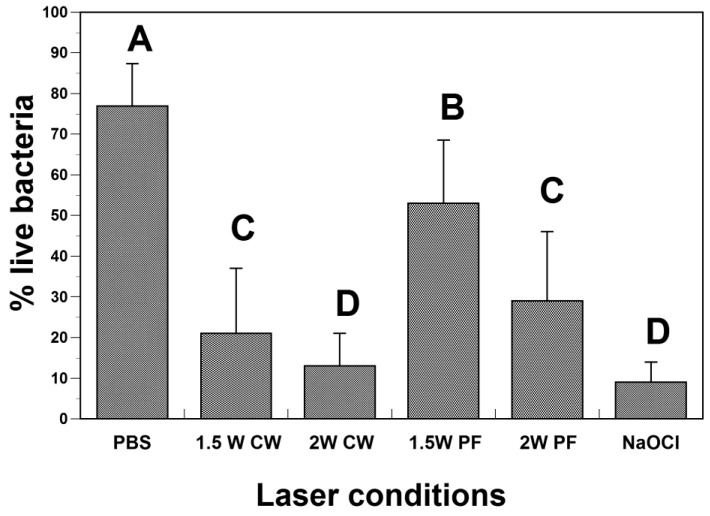
Bacterial survival after laser application. Letters represent statistical differences between groups (ANOVA, Tukey, *p* = 0.05). CW indicates continuous wave and PF indicates the pulse frequency in pulse mode.

After two weeks in the culture, the PBS control group showed an average of 77% viable bacteria. (Figure 3). For specimens exposed to sodium hypochlorite irrigation, the bacterial viability was significantly reduced, with only 8% of bacteria surviving after treatment. A marked decrease in bacterial viability was noted after laser irradiation in continuous mode at 1.5 W (21% of live bacteria) and 2 W (14% of live bacteria) (Figure 3). When the pulsed mode was applied, the reduction in bacterial viability was less, with a mean survival of 53% (1.5 PF, whereas 29% of bacteria survived after 2 W irradiation (Figure 3).

**Figure 4:**
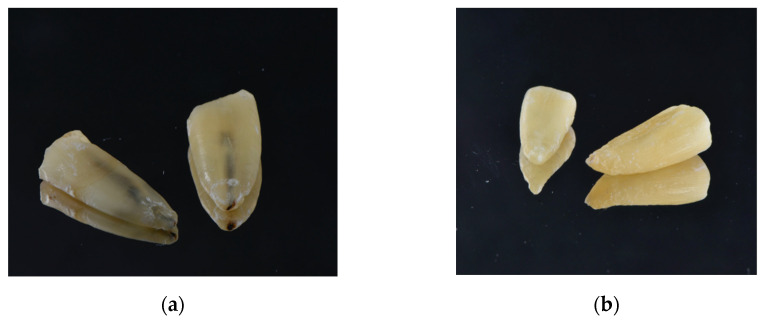
A morphological observation of specimens exposed to 2 W in continuous (**a**) or pulsed mode (**b**). A slight carbonization was observed inside the root canals exposed to 2 W continuous mode (**a**).

Mean temperature rise during laser irradiation is reported in Table 1. The mean temperature rise at 1.5 W in continuous mode was 7.1 °C, whereas the temperature increase at the apex of the root following 2 W irradiation was 7.9 °C. For the pulsed emission mode, the mean temperature increase was 4 ± 1.6 °C with the 1.5 W PF, and 5.9 ± 2.3 °C with 2 W PF. A morphological observation of specimens exposed to 2 W in continuous mode revealed the presence of a slight carbonization inside the root canal, but this pattern was not observed in the other groups (Figure 4).

## 4. Discussion

The present study’s results show that intracanal irradiation at 2 W in continuous mode effectively reduced the *E. faecalis* content inside root canals (86% reduction in viability) in a comparable manner to the NaOCl control group (*p* > 0.05), while the other laser conditions were considerably less effective (*p* < 0.05), independent of the emission mode selected (CW vs. PF) and despite the 80 s of irradiation applied. However, the cumulated irradiation time is much longer than that used by Gutknecht et al. who irradiated dentin disks for 32 s with an output power of 2.8 W and reported a 97% reduction in bacterial counts [53]. An average percentage decrease of 76% in *E. faecalis* count post-irradiation 4 × 5 s using an output power of 1.5 W was observed by Sarda et al. 2019 [54]. Another study by Borges et al. 2017 [45], indicated that irradiating dentin disks with a 970 nm diode (0.5 W for 4 s) only reduced bacterial viability by 68%. It is therefore suggested that increasing doses of 970 nm laser irradiation by multiplying irradiation periods does not necessarily improve root canal disinfection. However, the difference between our results and those previously reported by others may be related to the use of mature biofilms cultivated for 2 weeks inside root canals rather than dentin disks or roots contaminated within a shorter timeframe.

The present study used a biofilm model previously developed for evaluating procedures applied during endodontic irrigation by Provoost et al. 2022 [50]. This model relies on the use of mature biofilms of *E. faecalis* grown on root canal dentin, because *Enterococcus faecalis* are known to invade dentinal tubules [55] and mature biofilms were shown to be more resistant to antibacterial treatments [56]. In mature biofilms, nutrient-deprived cells maintain their viability while becoming more resistant to UV irradiation, heat, and treatment with sodium hypochlorite and other disinfecting solutions [57].

Fortunately, *E. faecalis* remains susceptible to heat, and exposure to temperatures of 60 °C and higher can irreversibly denature proteins and cellular structures, with both parameters being critical for its survival and replication [58]. However, the persistence of viable bacteria after laser application underscores the necessity for developing alternative or supplementary disinfection methods. Whereas the activation of sodium hypochlorite solutions with lasers [59,60] has shown promising results, the use of alternative irrigating solutions such as 2′-fucosyllactose and lacto-N-neotetraose remains to be confirmed [61].

Particular care was given to the sampling method, which relied on two separate steps to collect bacteria that may have survived after treatment. The first step involved a 30 K-File which was rotated in a reaming motion to scrub the canal walls and to retrieve bacteria potentially embedded in dentine, including at the apical level. In a second step, a sterile paper point was inserted at working length to pump out remaining fluids, then transferred into a sterile tube along with the K-File to generate a suspension used for assessing bacterial viability.

In the present study, temperature changes during lasing were measured close to the apex of the root, because dentin thickness is substantially lower at this level of the root and because this location has been previously used by others [46,62,63]. Previous research indicates that the critical temperature for bone and periodontal ligament damage is 10 °C above body temperature [64]. Our results show that this critical threshold was never reached, independent of the irradiation conditions. This agrees with Zou et al. 2020, who observed a temperature rise of 7.8 °C when an output power of 2.5 W was used [65]. Alfredo et al. 2008 showed much higher temperature rises (11 °C), but in specimens irradiated with an output power of 3 W in the continuous mode [46]. However, signs of carbonization were observed inside the root canals exposed to the 2 W continuous mode, thereby indicating that local excessive temperature rises may have occurred. Excessive temperature elevation after laser application have been shown to contribute to crack formation in root canal dentin [66], which may in turn lead to pain, damage to the cement layer and periodontal ligament, root resorption, and alveolar bone necrosis [67]. Although the purpose of the study was not to analyze carbonization in detail, but rather to identify its occurrence from a macroscopic perspective, it must be noted that structural alterations in dentine after irradiation were identified by others through scanning electron microscope analysis [46,68].

However, it must be noted that in an ex vivo setting, the teeth are disconnected from the human body, resulting in the loss of all systemic interactions. There is evidence showing that blood flow may serve as a heat absorber [69]. Also, there is no feedback from neuro-sensatory nerves regarding temperature changes, which may prevent the risks of over-heating in vivo.

The clinical implications of this study suggest the need for careful laser application, and the necessity for further studies to establish a clear protocol.

## 5. Conclusions

Within the limitations of this study, it can be concluded that 970 nm diode laser irradiation at a setting of 2 W CW has the potential to decrease the viability of mature *E. faecalis* biofilms within root canals, similar to sodium hypochlorite irrigation, without causing unacceptable temperature rises at the outer root surface. However, slight carbonization was observed at this setting. Therefore, the laser setting most suitable for clinical use is 1.5 W CW, since it did not result in any carbonization and therefore represents a good compromise between efficiency and safety.

## Figures and Tables

**Table 1:** Mean temperature rise after laser application. Letters represent statistical differences between groups (ANOVA, Tukey, *p* = 0.05).

Emission Mode	Power	∆ T °C	*p* < 0.05
Continuous mode	1.5 W	7.1 ± 2.8	A,a
	2 W	7.9 ± 3.1	A
Pulsed mode	1.5 W	4 ± 1.6	B,b
	2 W	5.9 ± 2.3	B

## Data Availability

The data presented in this study are available on request from the corresponding author.
